# Development of an anti-human EphA2 monoclonal antibody Ea_2_Mab-7 for multiple applications

**DOI:** 10.1016/j.bbrep.2025.101998

**Published:** 2025-04-01

**Authors:** Hiroyuki Satofuka, Hiroyuki Suzuki, Tomohiro Tanaka, Guanjie Li, Mika K. Kaneko, Yukinari Kato

**Affiliations:** Department of Antibody Drug Development, Tohoku University Graduate School of Medicine, 2-1 Seiryo-machi, Aoba-ku, Sendai, Miyagi, 980-8575, Japan

**Keywords:** EphA2, Monoclonal antibody, Cell-Based Immunization and Screening, Flow cytometry, Immunohistochemistry, Breast cancer

## Abstract

Ephrin type A receptor 2 (EphA2) binds to membrane-bound ligands, ephrin A1, A2, and A5, eliciting bidirectional signaling. This signaling regulates many physiological processes, such as tissue development, homeostasis, and regeneration. The dysregulation of the EphA2-ephrins axis contributes to various diseases, including cancers. The high expression of EphA2 is observed in various cancers, which promotes cancer malignancy, whereas its levels are relatively low in most normal adult tissues. Therefore, EphA2 is a promising target for cancer therapy. We developed anti-human EphA2 monoclonal antibodies in this study using the Cell-Based Immunization and Screening method. Among them, a clone Ea_2_Mab-7 (IgG_1_, κ) exhibited a high affinity and sensitivity in flow cytometry. The dissociation constant values of Ea_2_Mab-7 for CHO/EphA2 and MDA-MB-231 cells were determined as 6.2 ± 1.3 × 10^−9^ M and 1.6 ± 0.4 × 10^−9^ M, respectively. Furthermore, Ea_2_Mab-7 can detect endogenous EphA2 in Western blot and immunohistochemistry. Therefore, the Ea_2_Mab-7 is highly versatile for basic research and is expected to contribute to clinical applications, such as antibody therapy and tumor diagnosis.

## Highlights

• EphA2 is a promising target of antibody therapy for tumors.
• A novel anti-EphA2 monoclonal antibody, Ea_2_Mab-7, showed high affinity and specificity in flow cytometry.
• Ea_2_Mab-7 is also available for western blot and immunohistochemistry.
• Ea_2_Mab-7 is expected to aid the development of tumor diagnosis and therapy.

## 1 Introduction

Ephrin receptors (Eph) have a single transmembrane domain and are grouped into A and B categories based on their extracellular domains, which determine the binding affinity for ligands [1,2]. The interaction of Eph with the membrane-bound ephrin-A family ligands mediates communication between cells of the same or different types, and contact-dependent bidirectional (forward and reverse) signaling spreads from adjacent cells to neighboring cells [[3], [4], [5]]. The bidirectional signaling of the Eph system plays critical roles in tissue development, homeostasis, and regeneration.

EphA2 has been studied extensively in tumor development and progression [6]. Abundant expression of EphA2 has been reported in various cancers, such as prostate [7], lung [8], esophageal [9], colorectal [10], cervical [11], ovarian [12], skin [13], and breast cancers [14]. The expression of EphA2 is correlated with the progression of tumors [15]. EphA2 expression is associated with poor prognosis, elevated metastatic potential, and reduced patient survival [16,17]. Notably, the expression of EphA2 protein and mRNA was correlated with lymph node metastasis, clinical stage, and histologic grade of breast cancer [18]. Therefore, EphA2 is a promising target in cancer therapy because of its relatively low levels in most normal adult tissues [3].

Monoclonal antibody (mAb) therapy targeting EphA2 has been developed based on evidence that ligand stimulation, such as ephrin A1, is sufficient to induce EphA2 degradation through internalization [19]. For instance, anti-EphA2 mAbs (EA2 and B233) promoted EphA2 phosphorylation and degradation in cancer cells, and administration of EA2 mAb significantly decreased tumor growth in xenograft model [20]. In addition, a single-chain Fv of an anti-EphA2 mAb (D2) blocked the ligand interaction in COS-7 cells and induced apoptosis in the lymphoma cell line [21]. An anti-EphA2 mAb (SHM16) does not inhibit the interaction between ephrin A1 and EphA2 [22]. However, SHM16 stimulates the internalization of EphA2 and inhibits malignant features of melanoma A375 cells. A humanized anti-EphA2 mAb (DS-8895a) was developed as a therapeutic mAb with antibody-dependent cellular cytotoxicity (ADCC). Treatment with DS-8895a inhibited tumor growth of EphA2-positive human breast and gastric cancers in xenograft models [23]. Anti-EphA2 mAb-based immunotherapy using chimeric antigen receptor-T cells has also been developed and is undergoing clinical trials [24,25].

We have developed various mAbs against membrane proteins using the Cell-Based Immunization and Screening (CBIS) method [[26], [27], [28], [29], [30], [31], [32], [33]]. The mAbs obtained by this method are prone to recognize conformational epitopes and are suitable for flow cytometry. Furthermore, some of these mAbs are also available for Western blot, immunocytochemistry (ICC), and immunohistochemistry (IHC). This allows simultaneous contributions to the development of both therapy and diagnosis. Among the anti-EphA2 mAbs developed to date, SHM16 is known to recognize intact structure on the cell surface, while mAbs such as D4A2 are suitable only for Western blot and IHC [34,35]. Anti-EphA2 mAbs suitable for flow cytometry, Western blot, ICC, and IHC have not been reported (Supplementary Table 1). This study employed the CBIS method to generate a highly versatile anti-EphA2 mAb.

## 2 Materials and methods

### 2.1 Cell lines

Chinese hamster ovary (CHO)–K1, mouse myeloma P3X63Ag8U.1 (P3U1), human glioblastoma LN229, and human breast cancer MDA-MB-231 were obtained from American Type Culture Collection (ATCC, Manassas, VA, USA). The human pancreas cancer MIA PaCa-2 and colorectal cancer HCT-15 were obtained from the Cell Resource Center for Biomedical Research Institute of Development, Aging and Cancer at Tohoku University (Miyagi, Japan). The human lung cancer PC-10 was obtained from Immuno-Biological Laboratories Co., Ltd. (Gunma, Japan).

MDA-MB-231, MIA PaCa-2, HCT-15, LN229, and EphA2-overexpressed LN229 (LN229/EphA2) cells were maintained in Dulbecco's Modified Eagle Medium (DMEM) supplied with 100 U/mL penicillin, 100 μg/mL streptomycin, 0.25 μg/mL amphotericin B (Nacalai Tesque, Inc., Kyoto, Japan), and 10% heat-inactivated fetal bovine serum (FBS; Thermo Fisher Scientific, Inc., Waltham, MA, USA). CHO–K1, EphA2-overexpressed CHO-K1 (CHO/EphA2), P3U1, and PC-10 cells were maintained in Roswell Park Memorial Institute (RPMI)-1640 medium (Nacalai Tesque, Inc.) supplied with 100 U/mL penicillin, 100 μg/mL streptomycin, 0.25 μg/mL amphotericin B, and 10% heat-inactivated FBS. All the cells were cultured in a humidified incubator at 37C with 5% CO_2_.

### 2.2 Plasmid construction and establishment of stable transfectants

The gene encoding human *EPHA2* (NM_004431) was obtained from the RIKEN BioResource Research Center (Ibaraki, Japan). The open reading frame without signal sequence was subcloned into the pCAG-Ble vector (FUJIFILM Wako Pure Chemical Corporation, Osaka, Japan) using the In-Fusion HD Cloning Kit (Takara Bio, Inc., Shiga, Japan). The constructed vector was named pCAG-EphA2.

Using the Neon transfection system, the EphA2 gene-cloned plasmid was transfected into CHO–K1 and LN229 cells (Thermo Fisher Scientific, Inc.). Target gene-expressing transfectants were detected using an anti-EphA2 mAb, SHM16 (mouse IgG_2b_, κ, BioLegend, San Diego, CA, USA), and stable transfectants were established through cell sorting using a SH800 cell sorter (Sony Corporation, Tokyo, Japan). The established CHO/EphA2 and LN229/EphA2 cells were maintained in a medium containing 0.5 mg/mL of Zeocin (InvivoGen, San Diego, CA, USA).

### 2.3 Hybridoma production

The female BALB/cAJcl mice were purchased from CLEA Japan (Tokyo, Japan). All animal experiments were approved by the Animal Care and Use Committee of Tohoku University (Permit number: 2022MdA-001) and conducted under relevant guidelines and regulations to minimize animal suffering and distress in the laboratory. The mice were intraperitoneally immunized with LN229/EphA2 cells (1 × 10^8^ cells/injection) and Alhydrogel adjuvant 2% (InvivoGen). After three additional immunizations per week, a booster injection was administered two days before harvesting the spleen cells from immunized mice. The hybridomas were generated by fusing spleen cells with P3U1 cells using polyethylene glycol 1500 (Roche Diagnostics, Indianapolis, IN, USA). RPMI-1640 supplemented with hypoxanthine, aminopterin, and thymidine (HAT; Thermo Fisher Scientific, Inc.) was used to select hybridomas. The supernatants, which were negative for CHO–K1 cells and positive for CHO/EphA2 cells, were selected by flow cytometry using the SA3800 Cell Analyzer (Sony Corporation).

### 2.4 Flow cytometry

Cells were detached and harvested using 1 mM ethylenediaminetetraacetic acid (EDTA; Nacalai Tesque, Inc.) to prevent enzymatic degradation of cell surface proteins. The cells were washed with 0.1% bovine serum albumin (BSA) in phosphate-buffered saline (PBS, blocking buffer) and stained with mAbs by incubating for 30 min at 4°C. Subsequently, the cells were stained with Alexa Fluor 488-conjugated anti-mouse IgG (2000-fold dilution; Cell Signaling Technology, Inc., Danvers, MA, USA) for 30 min at 4°C. The data were collected using the SA3800 Cell Analyzer and analyzed using FlowJo software (BD Biosciences, Franklin Lakes, NJ, USA).

### 2.5 Determination of dissociation constant value using flow cytometry

CHO/EphA2 and MDA-MB-231 cells were treated with serially diluted Ea_2_Mab-7 and SHM16 (10–0.005 μg/mL). Subsequently, the cells were stained with Alexa Fluor 488-conjugated anti-mouse IgG (200-fold dilution) for 30 min at 4°C. The data were collected using the SA3800 Cell Analyzer and analyzed using FlowJo software. The fitting binding isotherms determined the dissociation constant (*K*_D_) values to built-in one-side binding models of GraphPad Prism 6 (GraphPad Software, Inc. La Jolla, CA, USA).

### 2.6 Western blot analysis

Whole cell lysates (10 μg of protein per lane) were separated into 5–20% polyacrylamide gels (FUJIFILM Wako Pure Chemical Corporation). The separated proteins were transferred onto polyvinylidene difluoride (PVDF) membranes (Merck KGaA, Darmstadt, Germany). The membranes were blocked with 4% skim milk (Nacalai Tesque, Inc.) in PBS containing 0.05% (v/v) Tween 20 (PBST; Nacalai Tesque, Inc.) and incubated with 1 μg/mL of Ea_2_Mab-7, SHM16, or an anti-β-actin mAb (clone AC-15; Sigma-Aldrich Corporation, St. Louis, MO, USA). Subsequently, they were incubated with anti-mouse IgG secondary Abs conjugated with horseradish peroxidase (1000-fold dilution; Agilent Technologies, Inc., Santa Clara, CA, USA). Chemiluminescence signals were developed with ImmunoStar LD (FUJIFILM Wako Pure Chemical Corporation) or Pierce ECL Plus Western Blotting Substrate (Thermo Fisher Scientific, Inc.) and detected using a Sayaca-Imager (DRC Co., Ltd., Tokyo, Japan).

### 2.7 Immunocytochemical analysis (ICC)

Cell blocks were prepared using iPGell (Genostaff Co., Ltd., Tokyo, Japan) and fixed with 4% paraformaldehyde phosphate buffer solution (FUJIFILM Wako Pure Chemical Corporation). The blocks were processed to make four μm thick paraffin-embedded cell sections that were directly autoclaved in a citrate buffer (pH 6.0; Nichirei Biosciences, Inc., Tokyo, Japan) for 20 min. These sections were blocked using the SuperBlock T20 Blocking Buffer (Thermo Fisher Scientific Inc.), incubated with Ea_2_Mab-7 (10 μg/mL) for 1 h at room temperature, and then treated with the Envision + Kit (Agilent Technologies Inc.) for 30 min. Color was developed using 3,3′-diaminobenzidine tetrahydrochloride (DAB; Agilent Technologies Inc.), and counterstaining was performed using hematoxylin (Merck KGaA, Darmstadt, Germany).

### 2.8 Immunohistochemical analysis (IHC)

A formalin-fixed paraffin-embedded (FFPE) breast invasive ductal carcinoma tissue array (Cat. No.: BR729) containing 71 cases was purchased (US Biomax, Inc., Rockville, MD, USA). The section was stained with Ea_2_Mab-7 (10 μg/mL) using BenchMark ULTRA PLUS (Roche Diagnostics, Indianapolis, IN, USA*).*

## 3 Results

### 3.1 Development of anti-human EphA2 mAbs

**Fig. 1:**
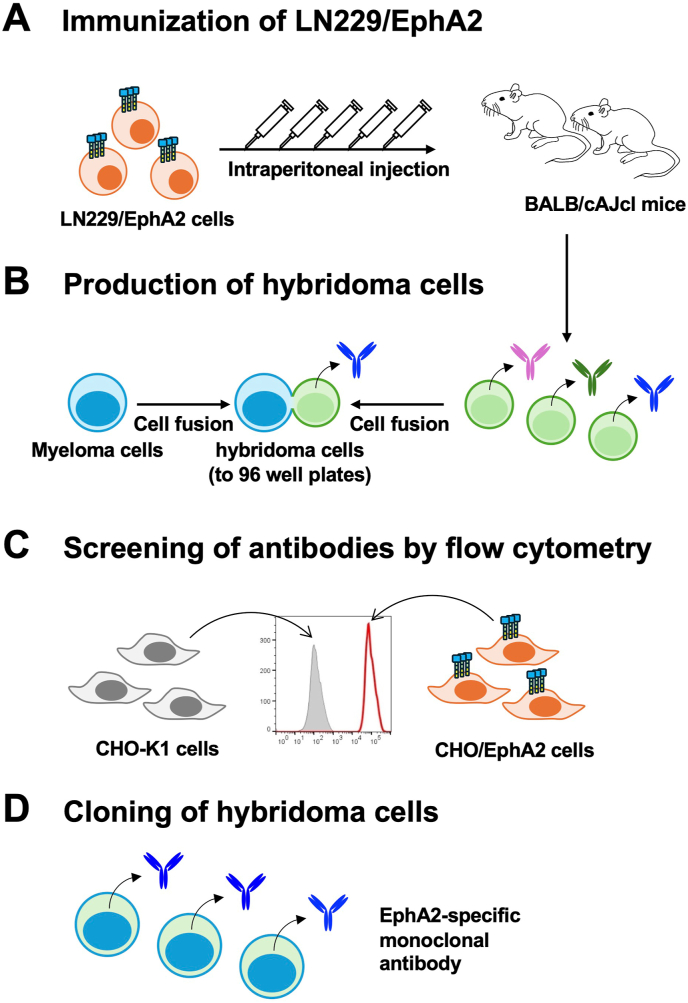
Schematic illustration of anti-EphA2 mAbs production. (A) LN229/EphA2 cells were intraperitoneally injected into BALB/cAJcl mice. (B) After immunization, spleen cells were collected and fused with P3U1 cells. (C) The supernatants of hybridoma cells were screened to obtain anti-EphA2 specific mAbs by flow cytometry using CHO/EphA2 and parental CHO–K1 cells. (D) Antigen-specific mAb-producing hybridoma cells were established by the limiting dilution method.

Two BALB/cAJcl mice were immunized with LN229/EphA2 cells, and the generated hybridomas were seeded into 96-well plates. After forming colonies, supernatants were collected and analyzed by flow cytometry-based high throughput screening to determine supernatants that were negative for CHO–K1 and positive for CHO/EphA2. Subsequently, anti-EphA2 mAb-producing hybridomas were cloned by limiting dilution. Finally, a clone Ea_2_Mab-7 (IgG_1_, κ) was established (Fig. 1).

### 3.2 Flow cytometry using Ea_2_Mab-7 and SHM16

**Fig. 2:**
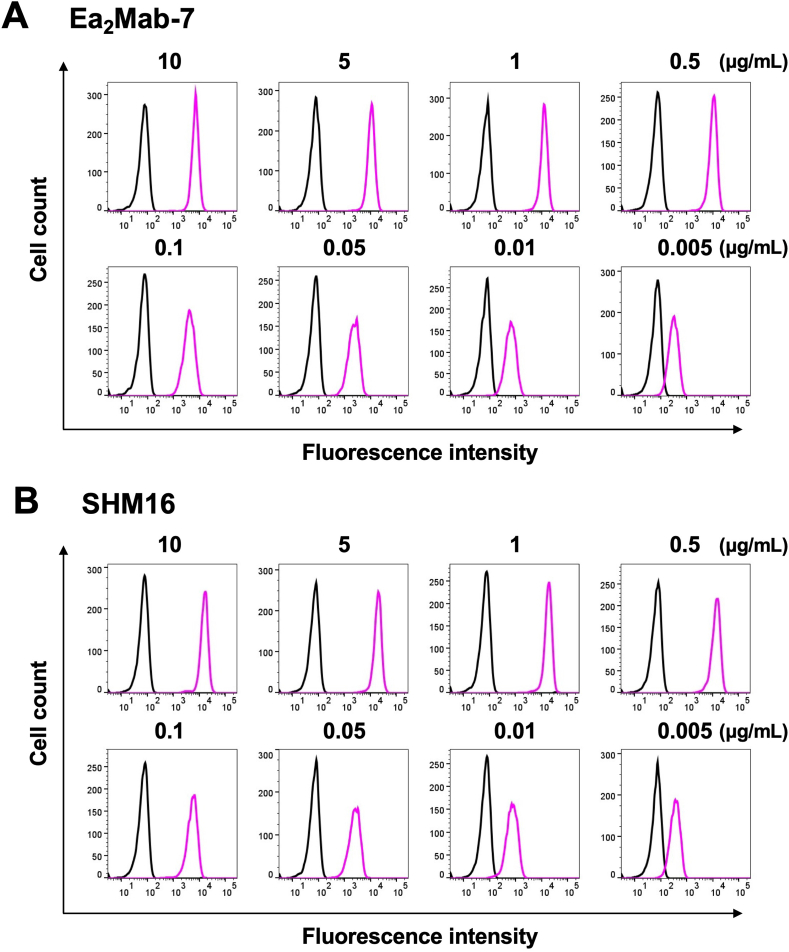
Flow cytometry of anti-EphA2 mAbs against CHO/EphA2 and CHO–K1 cells. CHO–K1 (black lines) and CHO/EphA2 (Purple lines) cells were treated with Ea_2_Mab-7 (A) and a commercially available anti-EphA2 mAb, SHM16 (B) at the indicated concentrations. The mAbs-treated cells were incubated with anti-mouse IgG conjugated with Alexa Fluor 488. The fluorescence data were subsequently collected using the SA3800 Cell Analyzer.

**Fig. 3:**
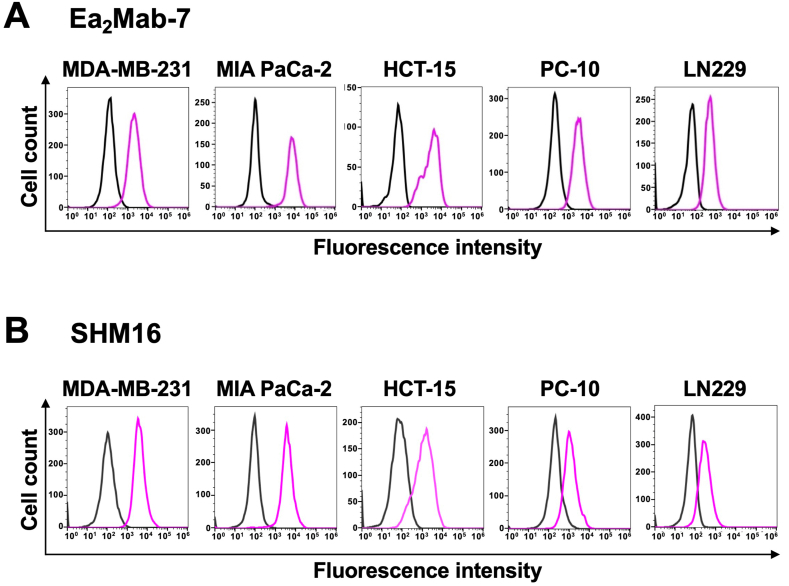
Flow cytometry of anti-EphA2 mAbs against endogenous EphA2 expressing cancer cells. MDA-MB-231, MIA PaCa-2, HCT-15, PC-10, and LN229 cells were treated with Ea_2_Mab-7 (A, Purple lines) and a commercially available anti-EphA2 mAb, SHM16 (B, Purple lines) at 10 μg/ml. The mAbs-treated cells were incubated with anti-mouse IgG conjugated with Alexa Fluor 488. The fluorescence data were subsequently collected using the SA3800 Cell Analyzer. The black line represents the negative control (blocking buffer).

We conducted flow cytometry using Ea_2_Mab-7 against CHO/EphA2 and CHO–K1 cells. Ea_2_Mab-7 recognized CHO/EphA2 cells in a dose-dependent manner, ranging from 10 to 0.005 μg/mL, but did not bind to CHO–K1 cells at any concentrations (Fig. 2A), indicating Ea_2_Mab-7 specifically recognizes EphA2 on the cell surface. A commercially available anti-EphA2 mAb (SHM16) exhibited a similar reactivity against CHO/EphA2 cells compared to Ea_2_Mab-7 (Fig. 2B). We next investigated the reactivity of Ea_2_Mab-7 against endogenously EphA2-expressing cell lines, MDA-MB-231, MIA PaCa-2, HCT-15, PC-10, and LN229. Ea_2_Mab-7 and SHM16 reacted with these cell lines at 10 μg/mL (Fig. 3).

### 3.3 Determination of *K*_D_ values of Ea_2_Mab-7 and SHM16 by flow cytometry

**Fig. 4:**
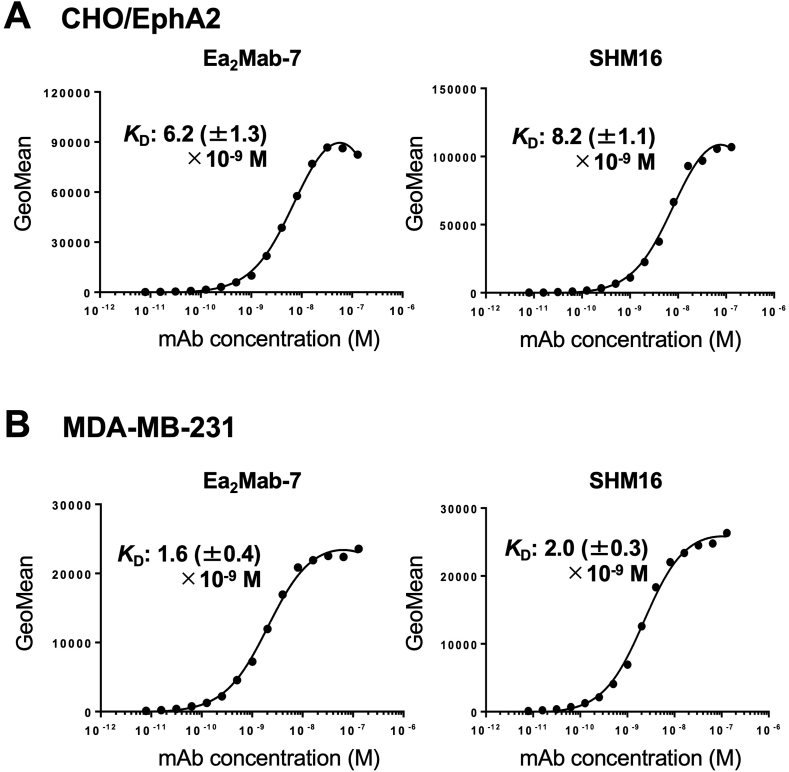
Measurement of binding affinity of Ea_2_Mab-7 and SHM16. CHO/EphA2 cells (A) and MDA-MB-231 cells (B) were stained with mAbs serially diluted at the indicated concentrations. The fluorescence data were subsequently collected using the SA3800 Cell Analyzer, followed by the calculation of the *K*_D_ using GraphPad PRISM 6. The average *K*_D_ values (± standard deviation) from three independent measurements were calculated by GraphPad PRISM 6 software. The representative graphs were shown.

**Supplementary Fig. 1:**
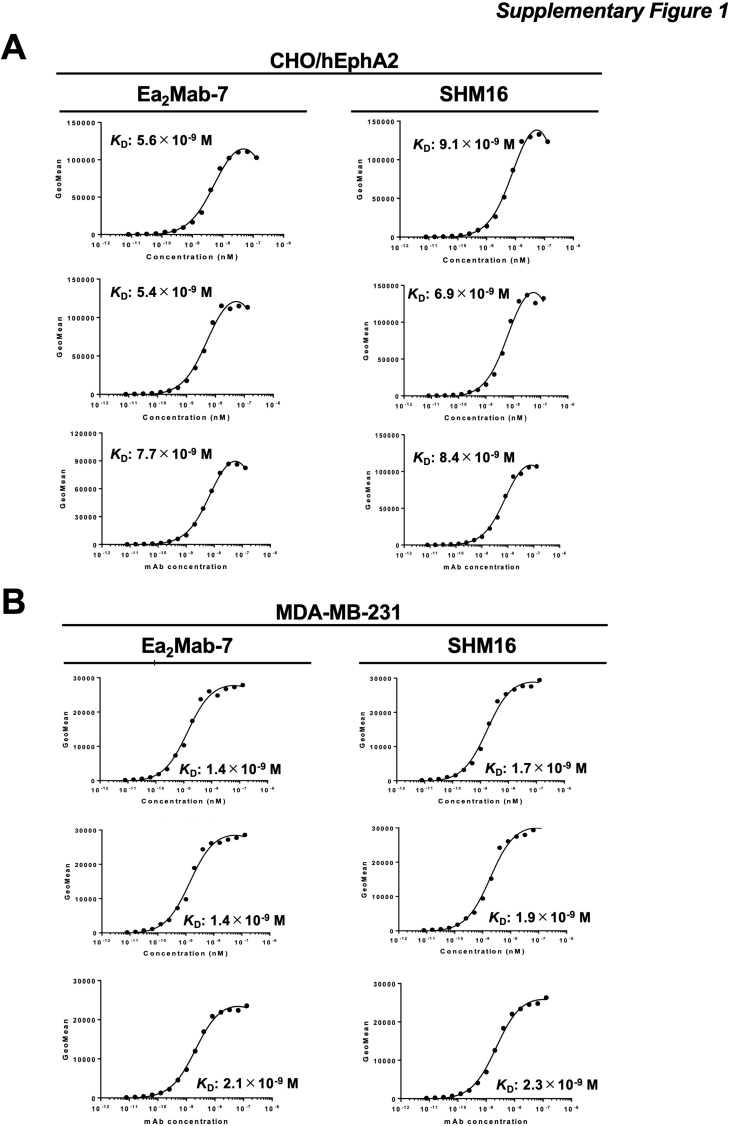
Three independent measurements were conducted to determine the binding affinity of Ea_2_Mab-7 and SHM16. CHO/EphA2 cells (A) and MDA-MB-231 cells (B) were stained with mAbs serially diluted at the indicated concentrations.

We performed flow cytometry to determine the binding affinity and calculated the *K*_D_ values of Ea_2_Mab-7 and SHM16. The *K*_D_ values obtained from three independent measurements of Ea_2_Mab-7 for CHO/EphA2 and MDA-MB-231 cells were 6.2 ± 1.3 × 10^−9^ M and 1.6 ± 0.4 × 10^−9^ M, respectively (Fig. 4 and Supplementary Fig. 1). The *K*_D_ values of SHM16 for CHO/EphA2 and MDA-MB-231 were 8.2 ± 1.1 × 10^−9^ M and 2.0 ± 0.3 × 10^−9^ M, respectively. These results indicated that the affinity of Ea_2_Mab-7 is slightly higher than that of SHM16.

### 3.4 Western blot analysis

**Fig. 5:**
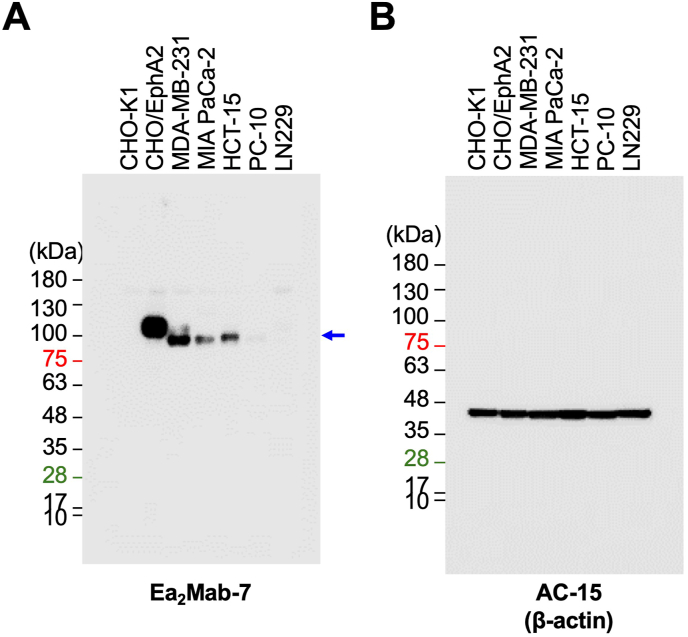
Western blot analysis using Ea_2_Mab-7. The cell lysate (10 μg/lane) of CHO–K1, CHO/EphA2, MDA-MB-231, MIA PaCa-2, HCT-15, PC-10, and LN229 cells were electrophoresed and transferred onto PVDF membranes. The membranes were incubated with 1 μg/mL of Ea_2_Mab-7 (A) and AC-15 (an anti-β-actin mAb) (B). The blue arrow indicates the estimated molecular weights of EphA2 (108 kDa).

**Supplementary Fig. 2:**
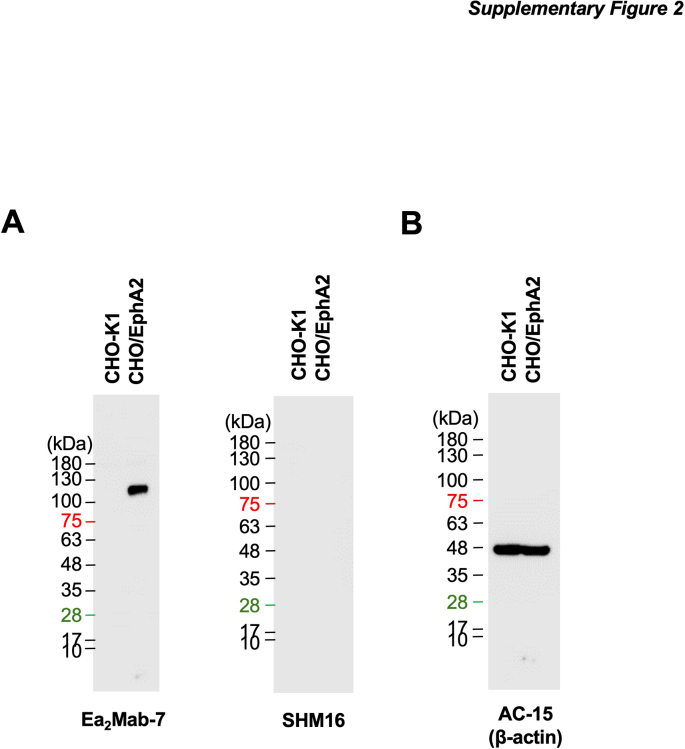
Western blot analysis using Ea_2_Mab-7 and SHM16. (A) The cell lysate (10 μg/lane) of CHO–K1 and CHO/EphA2 cells were electrophoresed and transferred onto PVDF membranes. (A) The membranes were incubated with a supernatant of Ea_2_Mab-7 hybridoma cells and 1 μg/mL of SHM16. (B) AC-15 (0.5 μg/mL) detected β-actin as loading control.

We next investigated whether Ea_2_Mab-7 is available for Western blot. Whole cell lysate of CHO–K1, CHO/EphA2, MDA-MB-231, MIA PaCa-2, HCT-15, PC-10, and LN229 were analyzed. Ea_2_Mab-7 showed a clear signal around the estimated molecular weight (108 kDa) of EphA2 in CHO/EphA2 (Supplementary Fig. 2A and Fig. 5B). In contrast, SHM16 was not available for Western blot (Supplementary Fig. 2A). Furthermore, Ea_2_Mab-7 is also capable of detecting endogenous EphA2 of MDA-MB-231, MIA PaCa-2, and HCT-15 (Fig. 5A). Weak signal was detected in PC-10 and LN229 (Fig. 5A).

### 3.5 ICC using Ea_2_Mab-7 in EphA2-overexpressed CHO–K1 cells

**Fig. 6:**
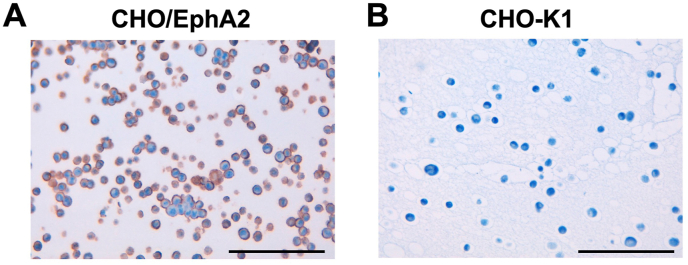
ICC of the paraffin-embedded cell sections of CHO–K1 and CHO/EphA2. The sections of CHO/EphA2 (A) and CHO–K1 (B) cells were treated with 10 μg/mL of Ea_2_Mab-7, followed by that with the Envision + Kit. Color was developed using DAB, and counterstaining was performed using hematoxylin. Scale bar = 100 μ m.

To investigate whether Ea_2_Mab-7 can be used for ICC analyses, paraffin-embedded CHO–K1 and CHO/EphA2 sections were stained with Ea_2_Mab-7. Apparent membranous staining by Ea_2_Mab-7 was observed in CHO/EphA2 (Fig. 6A) but not in CHO–K1 (Fig. 6B). These results indicate the usefulness of Ea_2_Mab-7 for detecting EphA2-positive cells in paraffin-embedded cell samples.

### 3.6 IHC using Ea_2_Mab-7 in breast cancer tissues

**Fig. 7:**
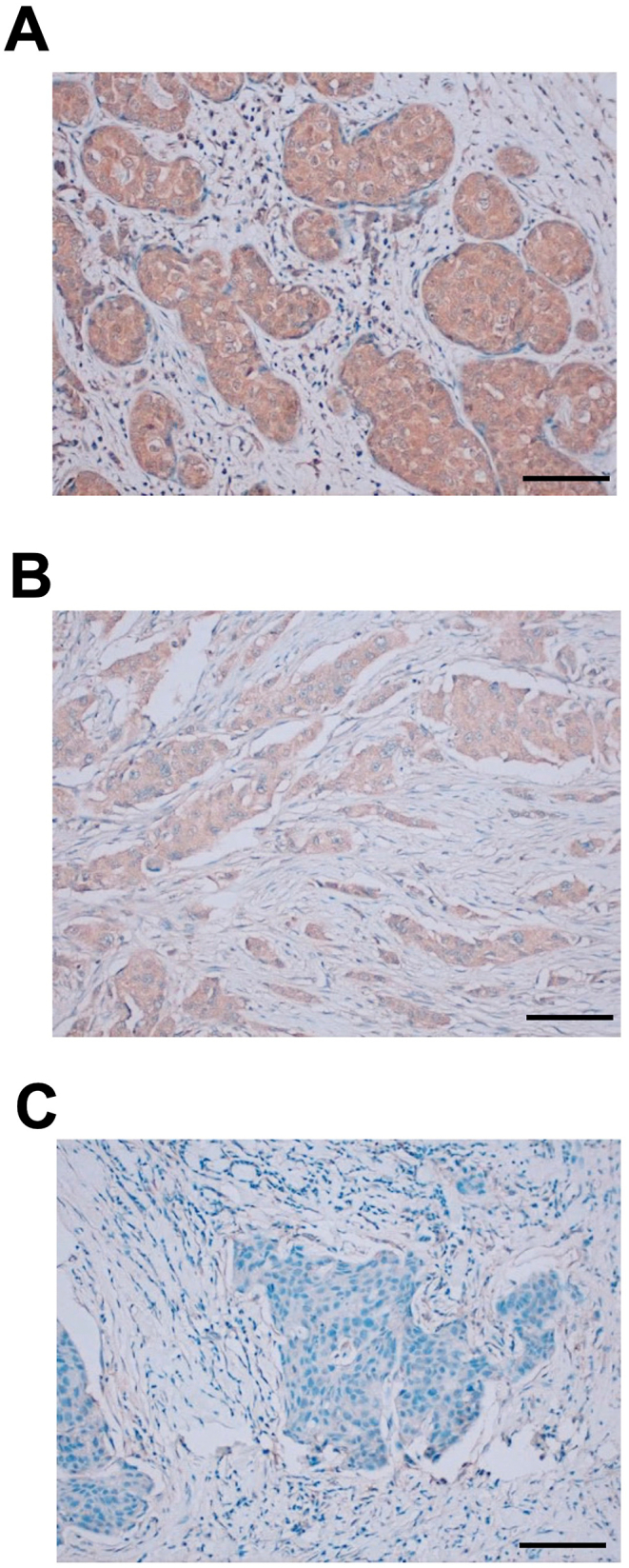
IHC using Ea_2_Mab-7 against breast cancer tissues. The sections of breast cancer tissue array (BR729) were treated with 10 μg/mL of Ea_2_Mab-7. The staining was carried out using VENTANA BenchMark Ultra. (A) EphA2-strong positive (2+); (B) EphA2-positive (1+); (C) EphA2-negative (0). Scale bar = 100 μm.

Next, the FFPE breast cancer tissue array was stained with Ea_2_Mab-7. The cytoplasmic pattern of EphA2 staining was observed. We classified the results into strong positive (2+, Fig. 7A), positive (1+, Fig. 7B), and negative (0, Fig. 7C). The staining results were summarized in Supplementary Table 2.

**Supplementary Fig. 3:**
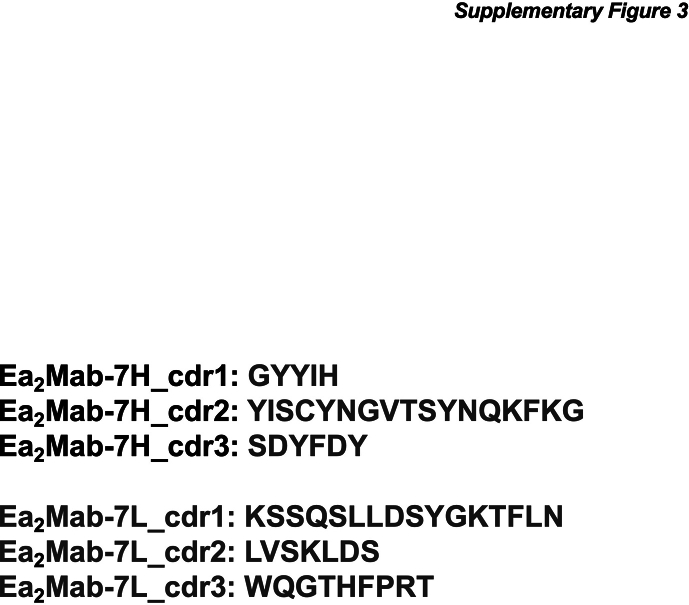
Amino acid sequence of complementarity-determining regions of Ea_2_Mab-7. The cDNA of Ea_2_Mab-7 variable regions was cloned, and the amino acid sequences of complementarity-determining regions (cdrs) were determined.

We cloned the cDNA of Ea_2_Mab-7 variable regions and showed amino acid sequences of complementarity-determining regions (Supplementary Fig. 3).

## 4 Discussion

In this study, we established a novel anti-EphA2 mAb, Ea_2_Mab-7, which is the first mAb suitable for various applications, including flow cytometry (Fig. 2, Fig. 3, Fig. 4), Western blot (Fig. 5), ICC (Fig. 6), and IHC (Fig. 7). We established twelve clones during the establishment of anti-EphA2 mAbs by the CBIS method. However, Ea_2_Mab-7 is the sole mAb suitable for all applications. Although SHM16 was established by the immunization of A375 melanoma cells into mice and developed as an agonistic mAb against EphA2 [22], SHM16 is not available to Western blot with the denatured form of EphA2, possibly due to its recognition of a structural epitope (Supplementary Fig. 1). In contrast, the D4A2 mAb is suitable for Western blot and IHC [34,35]; however, it is not useful for flow cytometry, possible due to its recognition of a liner epitope of EphA2. Therefore, identifying the epitope of Ea_2_Mab-7 is essential to reveal the structural basis of antigen recognition by Ea_2_Mab-7. We have developed the PA insertion for epitope mapping (PAMAP) and RIEDL insertion for epitope mapping (REMAP) to determine the epitope of mAbs [[36], [37], [38], [39], [40]]. The epitopes of anti-mouse CD39 mAb (C_39_Mab-1) could be determined using both PAMAP and REMAP methods [36]. Furthermore, the epitopes of anti-CD44 mAbs (C_44_Mab-5 and C_44_Mab-46) [37,38] and anti-EGFR mAbs (EMab-51 and EMab-134) [39,40] could be determined using the REMAP method. Therefore, further studies are required to determine the epitope of Ea_2_Mab-7. If Ea_2_Mab-7 recognizes the linear and non-glycosylated epitope, it may contribute to developing highly versatile mAbs to other Eph families using peptide immunization of the corresponding sequence.

Interaction between EphA2 and ephrin-As anchored on the plasma membranes of adjacent cells induces the large oligomeric complexes that mediate bidirectional signaling [5]. In contrast, the ephrin-A-independent non-canonical signaling of EphA2 plays a critical role in cancer progression [3]. The serine-threonine kinases of the RSK family [41] and AKT [42] can induce the phosphorylation of EphA2 (S897), which mediates oncogenic transformation [43], metabolic reprogramming [44], invasion [45], and resistance to therapies [8,46]. Furthermore, phosphorylated EphA2 (S897) has been shown to correlate with poor prognosis of breast cancer patients [47]. Therefore, anti-EphA2 mAbs can target cancer cells, which activate the non-canonical EphA2 signaling. To target the EphA2-positive cancer cells by Ea_2_Mab-7 (mouse IgG_1_), we should generate a class-switched mouse IgG_2a_ mAb from mouse IgG_1_. Furthermore, we previously produced defucosylated IgG_2a_ type mAbs to enhance the ADCC and *in vivo* antitumor effect in mouse xenograft models [48,49]. The class-switched and defucosylated type Ea_2_Mab-7 could contribute to treating EphA2-positive cancers in preclinical studies.

Focusing on the detailed signal intensities, different correlations are observed between applications. For instance, the Western blot signal did not wholly match the flow cytometry signal intensity (Fig. 3, Fig. 5A). Clear signals for CHO/EphA2, MDA-MB-231, MIA PaCa-2, and HCT-15 cells were detected in both flow cytometry and Western blot. However, weak or nearly undetectable signals were observed in PC-10 and LN229 cells in Western blot, while clear signals were detected in flow cytometry. Western blot shows the total amount of EphA2 expression in whole cells. At the same time, flow cytometry indicates the amount of EphA2 on the cell surface, suggesting that intracellular and extracellular levels of EphA2 differ depending on the cell line. This discrepancy may help explain the malignancy of cancer cells, as the abundant expression of EphA2 on the cell surface enables the triggering of non-canonical signaling that promotes cancer cell growth [3,19,50]. Additionally, compared to CHO/EphA2 cells, the signals of the endogenously expressed cell lines show a slightly smaller molecular weight in Western blot. The reason for this is not apparent at present, but post-translational modifications, including phosphorylation of EphA2 [41,51], may be contributing factors. Altogether, mAbs are suitable for various applications, such as Western blot and flow cytometry, and they provide significant advantages in estimating intracellular and extracellular protein levels and detecting protein processing.

Ea_2_Mab-7 can detect exogenous and endogenous EphA2 in ICC and IHC analysis (Fig. 6, Fig. 7). We could detect apparent membranous staining in the CHO/EphA2 section (Fig. 6). In contrast, IHC analysis of the breast cancer tissue array mainly showed cytoplasmic staining in most cases (Fig. 7 and Supplementary Table 2). This result was consistent with the previous reports using several anti-EphA2 Abs [35,52,53]. EphA2 was shown to receive the ligand-induced internalization [54]. Furthermore, the activation of receptor tyrosine kinase MET can induce the phosphorylation of EphA2 (S897) and endosomal internalization [55]. It would be interesting to investigate whether the cytoplasmic localization of EphA2 is involved in the malignant properties of cancer cells.

In conclusion, Ea_2_Mab-7 is a highly sensitive and versatile mAb for basic research and is expected to contribute to clinical applications, such as antibody therapy and tumor diagnosis.

## CRediT authorship contribution statement

**Hiroyuki Satofuka:** Writing – original draft, Investigation. **Hiroyuki Suzuki:** Investigation, Funding acquisition. **Tomohiro Tanaka:** Investigation, Funding acquisition. **Guanjie Li:** Investigation. **Mika K. Kaneko:** Funding acquisition, Conceptualization. **Yukinari Kato:** Writing – review & editing, Project administration, Funding acquisition, Conceptualization.

## Author disclosure statement

The authors have no conflict of interest.

## Funding information

This research was supported in part by 10.13039/100009619Japan Agency for Medical Research and Development (10.13039/100009619AMED) under Grant Numbers: JP24am0521010 (to Y.K.), JP24ama121008 (to Y.K.), JP23am0401013 (to Y.K.), JP24ama221339 (to Y.K.), JP24bm1123027 (to Y.K.), and JP24ck0106730 (to Y.K.), and by the 10.13039/501100001691Japan Society for the Promotion of Science (10.13039/501100001691JSPS) Grants-in-Aid for Scientific Research (10.13039/501100001691KAKENHI) grant nos. 24K11652 (to H.Satofuka), 22K06995 (to H.Suzuki), 24K18268 (to T.T.), and 22K07224 (to Y.K.).

## Declaration of competing interest

The authors declare the following financial interests/personal relationships which may be considered as potential competing interests: Yukinari Kato reports financial support was provided by 10.13039/100009619Japan Agency for Medical Research and Development. Hiroyuki Suzuki reports financial support was provided by 10.13039/501100001691Japan Society for the Promotion of Science. Hiroyuki Satofuka reports financial support was provided by 10.13039/501100001691Japan Society for the Promotion of Science. Tomohiro Tanaka reports financial support was provided by 10.13039/501100001691Japan Society for the Promotion of Science. If there are other authors, they declare that they have no known competing financial interests or personal relationships that could have appeared to influence the work reported in this paper.

## References

[1] Biao-xue R., Xi-guang C., Shuan-ying Y., Wei L., Zong-juan M. (2011). EphA2-dependent molecular targeting therapy for malignant tumors. Curr. Cancer Drug Targets.

[2] Tandon M., Vemula S.V., Mittal S.K. (2011). Emerging strategies for EphA2 receptor targeting for cancer therapeutics. Expert Opin. Ther. Targets.

[3] Pasquale E.B. (2024). Eph receptors and ephrins in cancer progression. Nat. Rev. Cancer.

[4] Pasquale E.B. (2005). Eph receptor signalling casts a wide net on cell behaviour. Nat. Rev. Mol. Cell Biol..

[5] Pasquale E.B. (2010). Eph receptors and ephrins in cancer: bidirectional signalling and beyond. Nat. Rev. Cancer.

[6] Zhou Y., Sakurai H. (2017). Emerging and diverse functions of the EphA2 noncanonical pathway in cancer progression. Biol. Pharm. Bull..

[7] Kurose H., Ueda K., Kondo R., Ogasawara S., Kusano H., Sanada S., Naito Y., Nakiri M., Nishihara K., Kakuma T., Akiba J., Igawa T., Yano H. (2019). Elevated expression of EPHA2 is associated with poor prognosis after radical prostatectomy in prostate cancer. Anticancer Res..

[8] Amato K.R., Wang S., Tan L., Hastings A.K., Song W., Lovly C.M., Meador C.B., Ye F., Lu P., Balko J.M., Colvin D.C., Cates J.M., Pao W., Gray N.S., Chen J. (2016). EPHA2 blockade overcomes acquired resistance to EGFR kinase inhibitors in lung cancer. Cancer Res..

[9] Miyazaki T., Kato H., Fukuchi M., Nakajima M., Kuwano H. (2003). EphA2 overexpression correlates with poor prognosis in esophageal squamous cell carcinoma. Int. J. Cancer.

[10] Martini G., Cardone C., Vitiello P.P., Belli V., Napolitano S., Troiani T., Ciardiello D., Della Corte C.M., Morgillo F., Matrone N., Sforza V., Papaccio G., Desiderio V., Paul M.C., Moreno-Viedma V., Normanno N., Rachiglio A.M., Tirino V., Maiello E., Latiano T.P., Rizzi D., Signoriello G., Sibilia M., Ciardiello F., Martinelli E. (2019). EPHA2 is a predictive biomarker of resistance and a potential therapeutic target for improving antiepidermal growth factor receptor therapy in colorectal cancer. Mol. Cancer Therapeut..

[11] Wu D., Suo Z., Kristensen G.B., Li S., Troen G., Holm R., Nesland J.M. (2004). Prognostic value of EphA2 and EphrinA-1 in squamous cell cervical carcinoma. Gynecol. Oncol..

[12] Lin Y.G., Han L.Y., Kamat A.A., Merritt W.M., Landen C.N., Deavers M.T., Fletcher M.S., Urbauer D.L., Kinch M.S., Sood A.K. (2007). EphA2 overexpression is associated with angiogenesis in ovarian cancer. Cancer.

[13] Mo J., Zhao X., Dong X., Liu T., Zhao N., Zhang D., Wang W., Zhang Y., Sun B. (2020). Effect of EphA2 knockdown on melanoma metastasis depends on intrinsic ephrinA1 level. Cell. Oncol..

[14] Youngblood V.M., Kim L.C., Edwards D.N., Hwang Y., Santapuram P.R., Stirdivant S.M., Lu P., Ye F., Brantley-Sieders D.M., Chen J. (2016). The ephrin-A1/EPHA2 signaling Axis regulates glutamine metabolism in HER2-positive breast cancer. Cancer Res..

[15] Wykosky J., Debinski W. (2008). The EphA2 receptor and ephrinA1 ligand in solid tumors: function and therapeutic targeting. Mol. Cancer Res..

[16] Kinch M.S., Moore M.B., Harpole D.H. (2003). Predictive value of the EphA2 receptor tyrosine kinase in lung cancer recurrence and survival. Clin. Cancer Res..

[17] Garcia-Monclús S., López-Alemany R., Almacellas-Rabaiget O., Herrero-Martín D., Huertas-Martinez J., Lagares-Tena L., Alba-Pavón P., Hontecillas-Prieto L., Mora J., de Álava E., Rello-Varona S., Giangrande P.H., Tirado O.M. (2018). EphA2 receptor is a key player in the metastatic onset of Ewing sarcoma. Int. J. Cancer.

[18] Zhou L., Lu X., Zhang B., Shi Y., Li Z. (2021). EphA2 as a new target for breast cancer and its potential clinical application. Int. J. Clin. Exp. Pathol..

[19] Xiao T., Xiao Y., Wang W., Tang Y.Y., Xiao Z., Su M. (2020). Targeting EphA2 in cancer. J. Hematol. Oncol..

[20] Coffman K.T., Hu M., Carles-Kinch K., Tice D., Donacki N., Munyon K., Kifle G., Woods R., Langermann S., Kiener P.A., Kinch M.S. (2003). Differential EphA2 epitope display on normal versus malignant cells. Cancer Res..

[21] Goldgur Y., Susi P., Karelehto E., Sanmark H., Lamminmäki U., Oricchio E., Wendel H.G., Nikolov D.B., Himanen J.P. (2014). Generation and characterization of a single-chain anti-EphA2 antibody. Growth Factors.

[22] Sakamoto A., Kato K., Hasegawa T., Ikeda S. (2018). An agonistic antibody to EPHA2 exhibits antitumor effects on human melanoma cells. Anticancer Res..

[23] Burvenich I.J., Parakh S., Gan H.K., Lee F.T., Guo N., Rigopoulos A., Lee S.T., Gong S., O'Keefe G.J., Tochon-Danguy H., Kotsuma M., Hasegawa J., Senaldi G., Scott A.M. (2016). Molecular imaging and quantitation of EphA2 expression in xenograft models with 89Zr-DS-8895a. J. Nucl. Med..

[24] Yi Z., Prinzing B.L., Cao F., Gottschalk S., Krenciute G. (2018). Optimizing EphA2-CAR T cells for the adoptive immunotherapy of glioma. Mol Ther Methods Clin Dev.

[25] Li N., Liu S., Sun M., Chen W., Xu X., Zeng Z., Tang Y., Dong Y., Chang A.H., Zhao Q. (2018). Chimeric antigen receptor-modified T cells redirected to EphA2 for the immunotherapy of non-small cell lung cancer. Transl Oncol.

[26] Asano T., Nanamiya R., Takei J., Nakamura T., Yanaka M., Hosono H., Tanaka T., Sano M., Kaneko M.K., Kato Y. (2021). Development of anti-mouse CC chemokine receptor 3 monoclonal antibodies for flow cytometry. Monoclon. Antibodies Immunodiagn. Immunother..

[27] Nanamiya R., Takei J., Asano T., Tanaka T., Sano M., Nakamura T., Yanaka M., Hosono H., Kaneko M.K., Kato Y. (2021). Development of anti-human CC chemokine receptor 9 monoclonal antibodies for flow cytometry. Monoclon. Antibodies Immunodiagn. Immunother..

[28] Nanamiya R., Suzuki H., Kaneko M.K., Kato Y. (2023). Development of an anti-EphB4 monoclonal antibody for multiple applications against breast cancers. Monoclon. Antibodies Immunodiagn. Immunother..

[29] Saito M., Suzuki H., Tanaka T., Asano T., Kaneko M.K., Kato Y. (2022). Development of an anti-mouse CCR8 monoclonal antibody (C. Monoclon. Antibodies Immunodiagn. Immunother..

[30] Suzuki H., Tanaka T., Li G., Ouchida T., Kaneko M.K., Kato Y. (2024). Development of a sensitive anti-mouse CCR5 monoclonal antibody for flow cytometry. Monoclon. Antibodies Immunodiagn. Immunother..

[31] Tanaka T., Nanamiya R., Takei J., Nakamura T., Yanaka M., Hosono H., Sano M., Asano T., Kaneko M.K., Kato Y. (2021). Development of anti-mouse CC chemokine receptor 8 monoclonal antibodies for flow cytometry. Monoclon. Antibodies Immunodiagn. Immunother..

[32] Tateyama N., Asano T., Suzuki H., Li G., Yoshikawa T., Tanaka T., Kaneko M.K., Kato Y. (2022). Epitope mapping of anti-mouse CCR3 monoclonal antibodies using flow cytometry. Antibodies.

[33] Yoshida S., Kato T., Kanno N., Nishimura N., Nishihara H., Horiguchi K., Kato Y. (2017). Cell type-specific localization of Ephs pairing with ephrin-B2 in the rat postnatal pituitary gland. Cell Tissue Res..

[34] Yasuta Y., Kaminaka R., Nagai S., Mouri S., Ishida K., Tanaka A., Zhou Y., Sakurai H., Yokoyama S. (2024). Cooperative function of oncogenic MAPK signaling and the loss of Pten for melanoma migration through the formation of lamellipodia. Sci. Rep..

[35] Nikas I., Giaginis C., Petrouska K., Alexandrou P., Michail A., Sarantis P., Tsourouflis G., Danas E., Pergaris A., Politis P.K., Nakopoulou L., Theocharis S. (2022). EPHA2, EPHA4, and EPHA7 expression in triple-negative breast cancer. Diagnostics.

[36] Okada Y., Suzuki H., Tanaka T., Kaneko M.K., Kato Y. (2024). Epitope mapping of an anti-mouse CD39 monoclonal antibody using PA scanning and RIEDL scanning. Monoclon. Antibodies Immunodiagn. Immunother..

[37] Asano T., Kaneko M.K., Takei J., Tateyama N., Kato Y. (2021). Epitope mapping of the anti-CD44 monoclonal antibody (C(44)Mab-46) using the REMAP method. Monoclon. Antibodies Immunodiagn. Immunother..

[38] Asano T., Kaneko M.K., Kato Y. (2021). Development of a novel epitope mapping system: RIEDL insertion for epitope mapping method. Monoclon. Antibodies Immunodiagn. Immunother..

[39] Sano M., Kaneko M.K., Aasano T., Kato Y. (2021). Epitope mapping of an antihuman EGFR monoclonal antibody (EMab-134) using the REMAP method. Monoclon. Antibodies Immunodiagn. Immunother..

[40] Nanamiya R., Sano M., Asano T., Yanaka M., Nakamura T., Saito M., Tanaka T., Hosono H., Tateyama N., Kaneko M.K., Kato Y. (2021). Epitope mapping of an anti-human epidermal growth factor receptor monoclonal antibody (EMab-51) using the RIEDL insertion for epitope mapping method. Monoclon. Antibodies Immunodiagn. Immunother..

[41] Zhou Y., Yamada N., Tanaka T., Hori T., Yokoyama S., Hayakawa Y., Yano S., Fukuoka J., Koizumi K., Saiki I., Sakurai H. (2015). Crucial roles of RSK in cell motility by catalysing serine phosphorylation of EphA2. Nat. Commun..

[42] Miao H., Li D.Q., Mukherjee A., Guo H., Petty A., Cutter J., Basilion J.P., Sedor J., Wu J., Danielpour D., Sloan A.E., Cohen M.L., Wang B. (2009). EphA2 mediates ligand-dependent inhibition and ligand-independent promotion of cell migration and invasion via a reciprocal regulatory loop with Akt. Cancer Cell.

[43] Macrae M., Neve R.M., Rodriguez-Viciana P., Haqq C., Yeh J., Chen C., Gray J.W., McCormick F. (2005). A conditional feedback loop regulates Ras activity through EphA2. Cancer Cell.

[44] Harly C., Joyce S.P., Domblides C., Bachelet T., Pitard V., Mannat C., Pappalardo A., Couzi L., Netzer S., Massara L., Obre E., Hawchar O., Lartigue L., Claverol S., Cano C., Moreau J.F., Mahouche I., Soubeyran I., Rossignol R., Viollet B., Willcox C.R., Mohammed F., Willcox B.E., Faustin B., Déchanet-Merville J. (2021). Human γδ T cell sensing of AMPK-dependent metabolic tumor reprogramming through TCR recognition of EphA2. Sci Immunol.

[45] Koshikawa N., Hoshino D., Taniguchi H., Minegishi T., Tomari T., Nam S.O., Aoki M., Sueta T., Nakagawa T., Miyamoto S., Nabeshima K., Weaver A.M., Seiki M. (2015). Proteolysis of EphA2 converts it from a tumor suppressor to an oncoprotein. Cancer Res..

[46] Chen Z., Liu Z., Zhang M., Huang W., Li Z., Wang S., Zhang C., Dong B., Gao J., Shen L. (2019). EPHA2 blockade reverses acquired resistance to afatinib induced by EPHA2-mediated MAPK pathway activation in gastric cancer cells and avatar mice. Int. J. Cancer.

[47] Mitra D., Bhattacharyya S., Alam N., Sen S., Mitra S., Mandal S., Vignesh S., Majumder B., Murmu N. (2020). Phosphorylation of EphA2 receptor and vasculogenic mimicry is an indicator of poor prognosis in invasive carcinoma of the breast. Breast Cancer Res. Treat..

[48] Ishikawa K., Suzuki H., Ohishi T., Nakamura T., Yanaka M., Li G., Tanaka T., Ohkoshi A., Kawada M., Kaneko M.K., Katori Y., Kato Y. (2024). Antitumor activities of anti-CD44 monoclonal antibodies in mouse xenograft models of esophageal cancer. Oncol. Rep..

[49] Ishikawa K., Suzuki H., Ohishi T., Li G., Tanaka T., Kawada M., Ohkoshi A., Kaneko M.K., Katori Y., Kato Y. (2024). Anti-CD44 variant 10 monoclonal antibody exerts antitumor activity in mouse xenograft models of oral squamous cell carcinomas. Int. J. Mol. Sci..

[50] Cioce M., Fazio V.M. (2021). EphA2 and EGFR: friends in life, partners in crime. Can EphA2 Be a predictive biomarker of response to anti-EGFR agents?. Cancers.

[51] Kaminskyy V.O., Haag P., Novak M., Vegvari A., Arapi V., Lewensohn R., Viktorsson K. (2021). EPHA2 interacts with DNA-PK(cs) in cell nucleus and controls ionizing radiation responses in non-small cell lung cancer cells. Cancers.

[52] Lévêque R., Corbet C., Aubert L., Guilbert M., Lagadec C., Adriaenssens E., Duval J., Finetti P., Birnbaum D., Magné N., Chopin V., Bertucci F., Le Bourhis X., Toillon R.A. (2019). ProNGF increases breast tumor aggressiveness through functional association of TrkA with EphA2. Cancer Lett..

[53] Li Y., Peng Q., Wang L. (2023). EphA2 as a phase separation protein associated with ferroptosis and immune cell infiltration in colorectal cancer. Aging (Albany NY).

[54] Boissier P., Chen J., Huynh-Do U. (2013). EphA2 signaling following endocytosis: role of Tiam1. Traffic.

[55] Marco S., Neilson M., Moore M., Perez-Garcia A., Hall H., Mitchell L., Lilla S., Blanco G.R., Hedley A., Zanivan S., Norman J.C. (2021). Nuclear-capture of endosomes depletes nuclear G-actin to promote SRF/MRTF activation and cancer cell invasion. Nat. Commun..

