# Supplementary material for: Development of an anti-human EphA2 monoclonal antibody Ea2Mab-7 for multiple applications
Source: Biochem Biophys Rep. 2025 Apr 1;42:101998. doi: 10.1016/j.bbrep.2025.101998 (PMC11999297; doi:10.1016/j.bbrep.2025.101998)
Supplement: Multimedia component 1 [file mmc1.docx]

Supplementary Table 1 Anti-EphA2 monoclonal antibodies

| Clone | Host | Subclass | FCM | *K*_D_ (M)* | WB | IHC |
| --- | --- | --- | --- | --- | --- | --- |
| Ea_2_Mab-7 | Mouse | IgG_1_, κ | + | 7.7×10^-9^ [CHO/EphA2] | + | + |
|  |  |  |  | 2.1×10^-9^  [MDA-MB-231] |  |  |
| SHM16 | Mouse | IgG_2b_, κ | + | 8.4×10^-9^ [CHO/EphA2] | - | n. d. |
|  |  |  |  | 2.3×10^-9^  [MDA-MB-231] |  |  |
| D4A2 | Rabbit | IgG | n. d. | n. d. | + | + |
| 1C11A12 | Mouse | IgG_1_, κ | n. d. | n. d. | + | + |
| MAB3035 | Mouse | IgG_2a_ | + | n. d. | + | + |

n. d.: not determined

*The *K*_D_ values of Ea_2_Mab-7 and SHM16 were determined in this study.
