# Supplementary material for: Development of an anti-human EphA2 monoclonal antibody Ea2Mab-7 for multiple applications
Source: Biochem Biophys Rep. 2025 Apr 1;42:101998. doi: 10.1016/j.bbrep.2025.101998 (PMC11999297; doi:10.1016/j.bbrep.2025.101998)
Supplement: Multimedia component 2 [file mmc2.docx]

Supplementary Table 2 Breast invasive ductal carcinoma tissue microarray (BR729)

| No. | Age | Pathology diagnosis | TNM | Stage | HER2 | Ea_2_Mab-7 |
| --- | --- | --- | --- | --- | --- | --- |
| 1 | 39 | Invasive ductal carcinoma | T2N0M0 | IIA | 2+ | 1+ |
| 2 | 36 | Invasive ductal carcinoma | T2N0M0 | IIA | 2+ | 2+ |
| 3 | 54 | Invasive ductal carcinoma | T1N0M0 | IA | 2+ | 1+ |
| 4 | 60 | Invasive ductal carcinoma | T2N0M0 | IIA | 2+ | 2+ |
| 5 | 46 | Invasive ductal carcinoma | T1N0M0 | IA | 2+ | 2+ |
| 6 | 53 | Invasive ductal carcinoma | T2N1M0 | IIB | 2+ | 2+ |
| 7 | 65 | Invasive ductal carcinoma | T2N1M0 | IIB | 1+ | 1+ |
| 8 | 31 | Invasive ductal carcinoma | T2N0M0 | IIA | 2+ | 1+ |
| 9 | 60 | Invasive ductal carcinoma | T2N0M0 | IIA | 2+ | 1+ |
| 10 | 43 | Invasive ductal carcinoma | T1N0M0 | IA | * | 1+ |
| 11 | 46 | Invasive ductal carcinoma | T2N1M0 | IIB | 2+ | 2+ |
| 12 | 58 | Invasive ductal carcinoma | T3N0M0 | IIB | 2+ | 2+ |
| 13 | 27 | Invasive ductal carcinoma | T2N0M0 | IIA | 2+ | 2+ |
| 14 | 38 | Invasive ductal carcinoma | T3N1M0 | IIIA | 2+ | 2+ |
| 15 | 59 | Invasive ductal carcinoma | T2N0M0 | IIA | 1+ | 2+ |
| 16 | 52 | Invasive ductal carcinoma | T2N1M0 | IIB | 3+ | 2+ |
| 17 | 57 | Invasive ductal carcinoma | T4N2M0 | IIIB | 3+ | 2+ |
| 18 | 38 | Invasive ductal carcinoma | T2N1M0 | IIB | 3+ | 2+ |
| 19 | 50 | Invasive ductal carcinoma | T2N0M0 | IIA | 2+ | 0 |
| 20 | 51 | Invasive ductal carcinoma | T3N1M0 | IIIA | 2+ | 2+ |
| 21 | 45 | Invasive ductal carcinoma | T1N0M0 | IA | 0 | 1+ |
| 22 | 64 | Invasive ductal carcinoma | T2N0M0 | IIA | 2+ | 2+ |
| 23 | 59 | Invasive ductal carcinoma | T2N2M0 | IIIA | 1+ | 1+ |
| 24 | 44 | Invasive ductal carcinoma | T2N0M0 | IIA | 2+ | 2+ |
| 25 | 39 | Invasive ductal carcinoma | T2N0M0 | IIA | 2+ | 2+ |
| 26 | 47 | Invasive ductal carcinoma | T2N1M0 | IIB | 2+ | 2+ |
| 27 | 50 | Invasive ductal carcinoma | T2N0M0 | IIA | 3+ | 1+ |
| 28 | 70 | Invasive ductal carcinoma | T2N0M0 | IIA | 3+ | 1+ |
| 29 | 50 | Invasive ductal carcinoma | T2N1M0 | IIB | 3+ | 1+ |
| 30 | 57 | Invasive ductal carcinoma | T2N0M0 | IIA | 0 | 1+ |
| 31 | 47 | Invasive ductal carcinoma | T2N0M0 | IIA | 2+ | 2+ |
| 32 | 57 | Invasive ductal carcinoma | T2N0M0 | IIA | 2+ | 1+ |
| 33 | 47 | Invasive ductal carcinoma | T2N0M0 | IIA | 0 | 1+ |
| 34 | 44 | Invasive ductal carcinoma | T2N1M0 | IIB | 3+ | 2+ |
| 35 | 45 | Invasive ductal carcinoma | T3N1M0 | IIB | 2+ | 2+ |
| 36 | 46 | Invasive ductal carcinoma | T2N0M0 | IIA | 3+ | 2+ |
| 37 | 66 | Invasive ductal carcinoma | T3N0M0 | IIB | 2+ | 1+ |
| 38 | 46 | Invasive ductal carcinoma | T3N0M0 | IIB | 2+ | 1+ |
| 39 | 57 | Invasive ductal carcinoma | T2N0M0 | IIA | 2+ | 2+ |
| 40 | 45 | Invasive ductal carcinoma | T2N0M0 | IIA | * | 2+ |
| 41 | 52 | Invasive ductal carcinoma | T4N1M0 | IIIB | 2+ | 1+ |
| 42 | 52 | Invasive ductal carcinoma | T2N2M0 | IIIA | 0 | 1+ |
| 43 | 40 | Invasive ductal carcinoma | T4N0M0 | IIIB | 2+ | 1+ |
| 44 | 52 | Invasive ductal carcinoma | T4N1M0 | IIIB | 2+ | 1+ |
| 45 | 54 | Invasive ductal carcinoma | T3N0M0 | IIB | 3+ | 2+ |
| 46 | 62 | Invasive ductal carcinoma | T2N0M0 | IIA | 2+ | 2+ |
| 47 | 49 | Invasive ductal carcinoma | T2N1M0 | IIB | 0 | 2+ |
| 48 | 54 | Invasive ductal carcinoma | T3N0M0 | IIB | 2+ | 2+ |
| 49 | 51 | Invasive ductal carcinoma | T4N1M0 | IIIB | 0 | 1+ |
| 50 | 42 | Invasive ductal carcinoma | T3N0M0 | IIB | 3+ | 2+ |
| 51 | 61 | Invasive ductal carcinoma | T3N1M0 | IIIA | 3+ | 2+ |
| 52 | 49 | Invasive ductal carcinoma | T2N2M0 | IIIA | 0 | 1+ |
| 53 | 50 | Invasive ductal carcinoma | T3N0M0 | IIB | 2+ | 2+ |
| 54 | 42 | Invasive ductal carcinoma | T3N0M0 | IIB | 3+ | 1+ |
| 55 | 35 | Invasive ductal carcinoma | T4N1M0 | IIIB | 2+ | 1+ |
| 56 | 46 | Invasive ductal carcinoma | T2N1M0 | IIB | 2+ | 1+ |
| 57 | 40 | Invasive ductal carcinoma | T2N1M0 | IIB | 2+ | 2+ |
| 58 | 67 | Invasive ductal carcinoma | T2N0M0 | IIA | 0 | 2+ |
| 59 | 49 | Invasive ductal carcinoma | T4N2M0 | IIIB | 2+ | 1+ |
| 60 | 63 | Invasive ductal carcinoma | T2N0M0 | IIA | 2+ | 2+ |
| 61 | 52 | Invasive ductal carcinoma | T2N0M0 | IIA | 3+ | 2+ |
| 62 | 64 | Invasive ductal carcinoma | T3N1M0 | IIB | 3+ | 2+ |
| 63 | 37 | Invasive ductal carcinoma | T4N2M0 | IIIB | 2+ | 1+ |
| 64 | 39 | Invasive ductal carcinoma | T2N2M0 | IIIA | 3+ | 1+ |
| 65 | 33 | Invasive ductal carcinoma | T2N0M0 | IIA | 0 | 1+ |
| 66 | 66 | Invasive ductal carcinoma | T2N0M0 | IIA | 2+ | 1+ |
| 67 | 73 | Invasive ductal carcinoma | T4N0M0 | IIIB | 2+ | 1+ |
| 68 | 62 | Invasive ductal carcinoma | T4N0M0 | IIIB | 2+ | 1+ |
| 69 | 58 | Invasive ductal carcinoma | T2N0M0 | IIA | 2+ | 2+ |
| 70 | 58 | Invasive ductal carcinoma | T2N0M0 | IIA | 2+ | 2+ |
| 71 | 52 | Invasive ductal carcinoma | T2N1M0 | IIB | 3+ | 1+ |
| 72 | 45 | Intraductal carcinoma | T2N0M0 | IIA | 1+ | 0 |

*, invalid core
