# Supplementary figures and images for: Development of an anti-human EphA2 monoclonal antibody Ea2Mab-7 for multiple applications
Source: Biochem Biophys Rep. 2025 Apr 1;42:101998. doi: 10.1016/j.bbrep.2025.101998 (PMC11999297; doi:10.1016/j.bbrep.2025.101998)

Figure 5

A

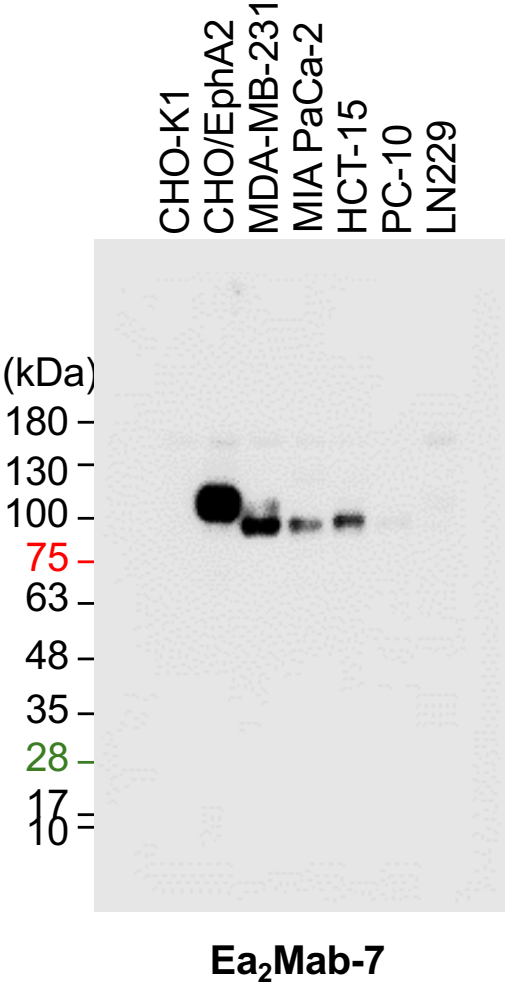

B

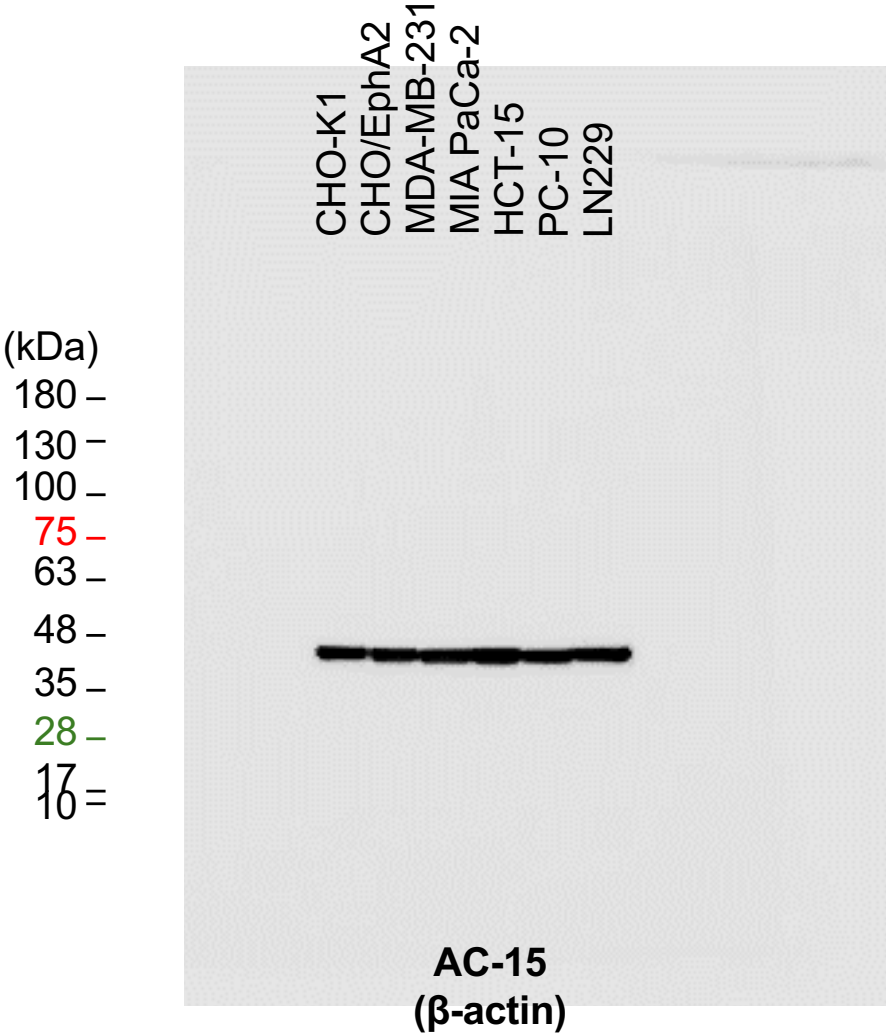

**A**

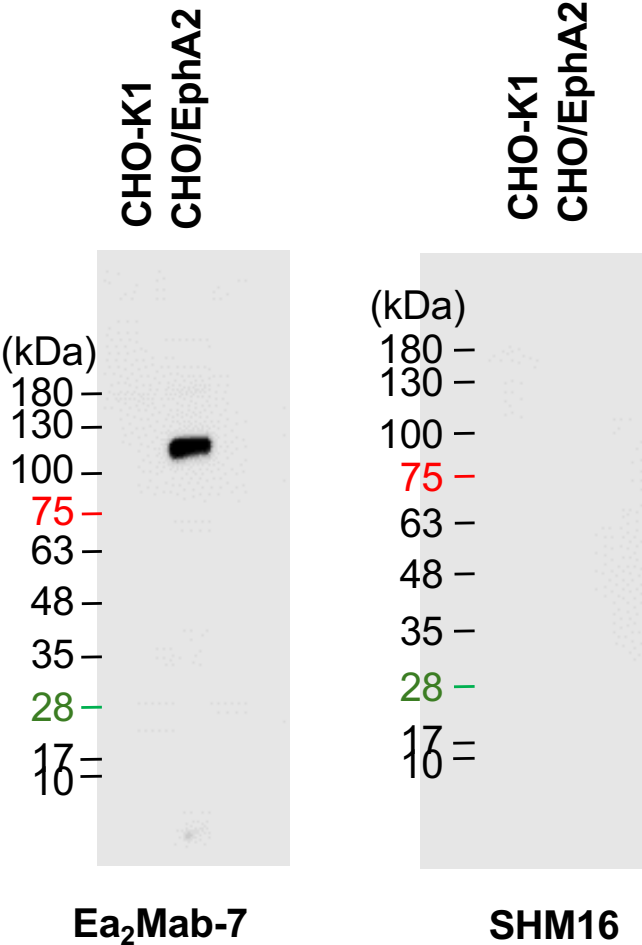

**B**

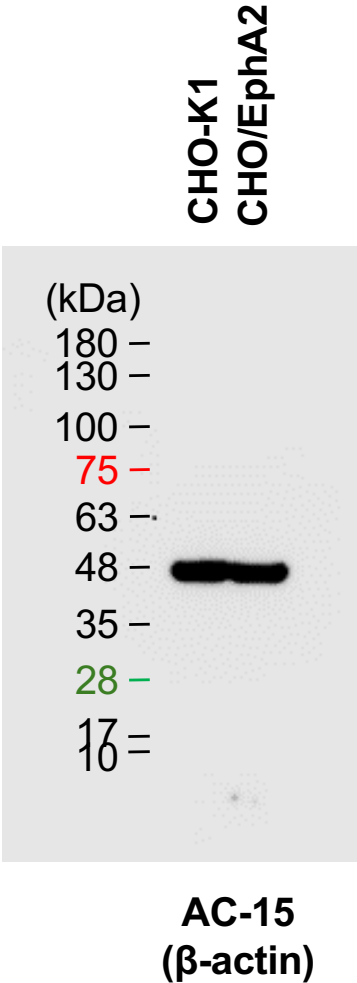

Supplement: Multimedia component 3 [file mmc3.pdf]
